# The Role of Early Programming and Early Nutrition on the Development and Progression of Celiac Disease: A Review

**DOI:** 10.3390/nu12113427

**Published:** 2020-11-08

**Authors:** Rafael Martín-Masot, Javier Diaz-Castro, Jorge Moreno-Fernandez, Víctor Manuel Navas-López, Teresa Nestares

**Affiliations:** 1 Pediatric Gastroenterology and Nutrition Unit, Hospital Regional Universitario de Málaga, 29010 Málaga, Spain; rafammgr@gmail.com (R.M.-M.); victor.navas@gmail.com (V.M.N.-L.); 2 Department of Physiology and Institute of Nutrition and Food Technology “José MataixVerdú”, Biomedical Research Centre, University of Granada, 18010 Granada, Spain; javierdc@ugr.es (J.D.-C.); jorgemf@ugr.es (J.M.-F.)

**Keywords:** celiac disease, early programming, perinatal nutrition

## Abstract

Experimental and epidemiological evidence has shown that modifications of the intrauterine environment can have deleterious consequences for individuals, expressed as an increased risk of suffering non-communicable pathologies in adult life, which is known as the hypothesis of the early origin of diseases or fetal programming. On the other hand, changes in gene expression patterns through epigenetic modifications can be the basis for long-term maintenance of the effects of fetal programming. In this sense, epigenetics comprises the study of intrauterine disturbances, which develop diseases in the adult, including celiac disease (CD). In addition, early feeding practices could influence the risk of CD development, such as breastfeeding timing and duration and age of gluten introduction in the diet. Gluten acts as a trigger for CD in genetically predisposed subjects, although approximately 30% of the world population has HLA DQ2 or DQ8, the prevalence of the disease is only 1–3%. It is not known what factors act to modify the risk of disease in genetically at-risk subjects. Taking into account all these considerations, the aim of the current review is to elucidate the role of early programming and the effect of early nutrition on the development and progression of CD. It is logical that attention has been paid to gluten as a key element in preventing the disease. However, there is no strong evidence in favor of the protective factor of breastfeeding, timing of introduction of gluten during lactation, and the development of CD. Diet, genetic risk, microbiota, and environmental interaction are possible triggers of the change in tolerance to an immune response to gluten, but large-scale cohort studies are needed. Emerging scientific concepts, such as epigenetics, may help us establish the role of these factors.

## 1. Introduction

The term early programming postulates that stimuli, environment, or insults during critical developmental periods can have an impact in the human lifespan [1]. In this sense, fetal life is one of the stages of greater plasticity, since at this stage, most of the organs and tissues are formed and developed. Experimental and epidemiological evidence has shown that modifications of the intrauterine environment can have deleterious consequences for individuals, expressed as an increased risk of suffering non-communicable pathologies in adult life, which is known as the hypothesis of the early origin of diseases or fetal programming [2].

Observational and cohort studies in England, Scandinavia, and India were the first to show the close association between low birth weight and the subsequent development of chronic diseases [3]. Barker et al. [4] described, in 16,000 subjects in England, that death rates from cardiovascular disease were halved in those who were born at normal weight compared to those who were small for gestational age. Furthermore, this association was maintained after adjusting for multiple variables, such as socioeconomic level, family history, and weight at the time of the study. Interestingly, a higher body mass index in adult life increased the strength of this association [5]. Other anthropometric indicators of impaired fetal growth, for example, weight, height, low abdominal circumference, and a high fetus–placental relationship, have been associated with the development of chronic disease in adult life [4,6].

In general, there are multiple factors that can alter birth weight, including maternal, placental, and fetal factors [7]. The main causes of fetal macrosomia include gestational diabetes, obesity, and maternal overnutrition [8]. The placenta is a fundamental organ during pregnancy since it participates in the transport of nutrients, in the immune response, in the synthesis of steroid and peptide hormones, and in the detoxification of substances harmful to the fetus. Therefore, an altered or poor placental function could be a central element in establishing an altered pattern of fetal development [9].

The concept of fetal programming involves a series of modifications in the structure and function of different tissues and organs, among which are a reduction or redistribution of different cell populations or an abnormal sensitivity of tissues to hormonal regulation [10]. On the other hand, changes in gene expression patterns through epigenetic modifications, such as DNA methylations, and post-translational modifications in histones and microRNAs, can be the basis for long-term maintenance of the effects of fetal programming [11]. Given the complexity of these processes, it is highly probable that there are multiple variables that affect the final result of fetal programming. Sexual dimorphism could be one of its main modifiers because male fetuses adapt differently to female fetuses in the face of unfavorable conditions during pregnancy [12]. Abnormal development during fetal life is now thought to contribute to the etiology of many functional and behavioral disorders that manifest throughout life. The intrauterine conditions in which the mammalian fetus develops (intrauterine environment) have an important role in regulating the function of its physiological systems later in life. Changes in the intrauterine availability of nutrients, oxygen, and hormones program tissue development and lead to abnormalities in adult cardiovascular and metabolic function in several species. Intrauterine changes of physiological systems occur at the gene, cell, tissue, organ, and system levels and cause permanent structural and functional changes, which can lead to overt disease [11].

However, the effect of early nutrition on long-term health does not occur only during the embryonic-fetal period but is prolonged in the first years of life. Celiac disease (CD) is a systemic disorder, caused by an immune reaction triggered by the ingestion of gluten and related proteins, which occurs in people who carry the DQ2 and/or DQ8 human leukocyte antigen (HLA) class II haplotypes, and it is characterized by a variable combination of high CD-specific antibody titers, an inflammatory enteropathy with a wide range of digestive and/or systemic symptoms [13,14,15]. Recent articles have shown that the HLA-DQ prevalence in the general world population is about 40–50% [16,17]. This pathological response generates severe atrophy of the intestinal villi and severe malabsorption of the nutrients [18]. Gluten causes an abnormal immune response in patients, generating autoantibodies transglutaminase type 2 (TG2) [19], and has a strong hereditary genetic component as testified by its high familial recurrence (~10–15%) and the high concordance of the disease among monozygotic twins (75–80%) [18]. In this sense, epigenetics comprises the study of intrauterine disturbances, which develop diseases in the adult, such as cancer, cardiovascular and metabolic diseases, and neurological, reproductive and immunological disorders [20], including also CD. In Sweden, a high increase of typical cases of CD was observed between 1984 and 1996 in infants younger than 2 years of age and it was hypothesized that it was due to changes induced in infant feeding [21,22], such as breastfeeding timing and duration and age at gluten introduction in the diet. Among atypical symptoms of CD, disorders of fertility, such as delayed menarche, early menopause, amenorrhea, or infertility, and pregnancy complications, such as recurrent abortions, intrauterine growth restriction (IUGR), small for gestational age (SGA) babies, low birth weight (LBW) babies, or preterm deliveries, must be factored [23]. The polyclonal fractions of anti-trans glutaminase (TG) antibodies are able to bind to trophoblast cells, inducing apoptotic damage. In addition to the anti-TG antibody-binding-mediated increase in trophoblast apoptosis, a significant decrease in matrix metalloproteases activity was observed, and this could be an indirect effect of the increase in trophoblast apoptosis [24], providing a pathogenic model of immune-mediated placental damage potentially occurring in vivo in women with active CD.

Therefore, it is biologically likely that breastfeeding at the time of gluten introduction in the diet increases the chance of developing oral tolerance for the major gluten antigens. Taking into account all these considerations, the aim of the current review was to elucidate the role of early programming on the development and progression of CD.

## 2. Metabolic Programming in Autoimmune Diseases

Genome-wide association studies (GWASs) have revolutionized the study of the genetic role of diseases, although the mechanisms have still not been completely elucidated due to the difficulty of assigning the biological meaning of many genetic variants [25]. Autoimmune inflammatory diseases, which reflect complex interactions between genetic variants and the environment, are important systems for the investigation of genetic diseases in humans [26]. The grouping of different autoimmune diseases in families suggests the existence of hereditary factors underlying common pathways of the disease, although the different clinical presentation and response to drugs differentiates them [27]. In 2015, Kai et al. [28] designed an algorithm to predict the association between a genetic variant and an autoimmune disease, concluding that most of the risk variants alter gene expression.

Thus, the prevalence of CD is higher in subjects suffering from other autoimmune diseases: (10 to 15%), type 1 diabetes (3 to 16%), Hashimoto’s thyroiditis (5%), or other autoimmune diseases (including autoimmune liver diseases, Sjögren’s syndrome, and IgA nephropathy), Down’s syndrome (5%), Turner’s syndrome (3%), and IgA deficiency (9%) [29,30,31,32,33,34]. Reasons for the concurrence among these autoimmune diseases include shared HLA genetic risk [35,36,37]. It is well known that the level of expression of HLA molecules on the cell surface is associated with the pathophysiology of autoimmune diseases and, currently, the focus of research is on studying the personalized prevention of autoimmune diseases modulating the HLA–ligand relationship [38].

The possible roles that prenatal and perinatal life events may play in disease development are known, and the fetal origin of immune-mediated diseases has also been investigated [39]. For some autoimmune diseases, such as type 1 diabetes, the importance of the intrauterine environment has been studied by comparing concordance rates in dizygotic and monozygotic twins [40]. Higher concordance rates between twins sharing the same proportion of genes as siblings could support mechanisms that operate in the uterus, but there are also more similarities in twins than siblings in the postnatal life. However, this approach has not been tested for CD, as far as we know.

Early nutrition can potentially alter future metabolic programming [41]. The available literature data show a growing interest and concern about the impact of both the timing and the modality of complementary feeding on the appearance of subsequent non-communicable diseases, including CD. The most plausible explanation is that these factors modify the expressiveness of certain genes, modifying the response of certain organs and tissues, and remodeling their structure and function. These epigenetic changes can be transmitted from one generation to the next, further highlighting the importance of the phenomenon of early metabolic programming.

The long-term health effects of breastfeeding, on both the prevention of autoimmune and metabolic diseases are known [42] and they fit into the early metabolic programming scheme. Not only the type and duration of breastfeeding are important, but also the timing of the introduction of complementary feeding and its characteristics. Most studies have focused on the period of breastfeeding, although time and composition can have similar subsequent effects. In this sense, timing is really important, because it is a period of rapid growth in which the body is susceptible to nutritional imbalances and there are rapid and marked changes in the diet with exposure to many new foods that could influence disease development through various mechanisms, including the effects of metabolic programming [43,44]. Current evidence recommends avoiding the introduction of solid foods before 4 months to avoid obesity and certain allergies [43]. However, in contrast to these studies, Lionetti et al. [45] showed that neither the delayed introduction of gluten nor breastfeeding modifies the risk of CD among at-risk infants, although the later introduction of gluten was associated with a delayed onset of disease.

High body mass index is also a risk factor. Current evidence shows that not only low birth weight predisposes infants to a higher risk of developing chronic diseases in adult life but also excessive fetal growth (macrosomia), as occurs in the offspring of obese or diabetic pregnant women [46,47].

## 3. Prenatal Conditions Associated with Celiac Disease

Maternal nutrition, together with environmental exposure (a wide range of both natural and synthetic substances, such as industrial and agricultural chemicals, plant and animal agents, naturally occurring metals, and bacteria-derived-compounds), can permanently affect fetal and neonatal gene expression through epigenetic mechanisms that lead to metabolic abnormalities that have important long-term effects on the health of the child [39,48].

Maternal obesity and maternal overnutrition can cause epigenetic alterations during pregnancy and these alterations can influence the fetal and neonatal phenotype, increasing the risk of metabolic disorders in later stages of life [49]. The maternal body mass index is positively correlated with systemic inflammation, including high levels of proinflammatory cytokines. Furthermore, an increase in these cytokines in the placenta has been shown to occur as a result of a high-fat diet. The high-fat maternal diet also causes insulin resistance through inflammatory changes in fetal adipose tissue. As a result of all these metabolic disturbances, excessive exposure to fetal lipids can affect fetal growth and development. Increased inflammation and blood lipids can have detrimental effects on the development of the liver, adipose tissue, brain, skeletal muscle, and pancreas, increasing the risk of metabolic disorders [50]. Dietary factors can affect genome function and gene expression during early life through folate-mediated single-carbon metabolism or transmethylation pathways. Alterations in gene expression during pregnancy can influence the fetal and neonatal phenotype [51].

On the other hand, pregnancy is considered as a factor that can unmask latent CD [52]. The gynecological and obstetric manifestations of CD are very varied. Among untreated celiac women, late menarche, premature births, early menopause, and difficulties for fertilization are more common [53]. However, a large population study found no differences in infertility between celiac women and the general population when obstetric outcomes were compared between women with and without CD [54]. In active CD, malabsorption can lead to a deficit of different nutrients, such as zinc, selenium, folic acid, or iron, among others. Zinc and selenium deficiency affect gonadotropin synthesis, so the gonadal axis can be disrupted, and folic acid deficiency can affect neural tube development in the fetus [55]. The severity of malnutrition is directly correlated with the frequency and severity of gynecological-obstetric disorders, and a gluten-free diet has shown similar results in women with CD as in the general population [56]. The presence of TG has been demonstrated in endometrial cells, as well as in stroma and trophoblastic cells [57]. It has been postulated that the binding of TG and anti-TG may affect endometrial angiogenesis and decidualization, and thus affect implantation [58].

Several studies reveal that women with CD have a shortening of their fertile life with a delay in menarche and an earlier menopause compared to healthy women [59,60]. Additionally, fertility problems are common in both men and women with CD and can be the first symptom of the disease. Other studies have indicated that there is a higher frequency of perinatal mortality and abortions among undiagnosed celiac women. These undiagnosed and untreated women are at higher risk of having low birth weight children than the general population [59,60].

In addition, as a consequence of CD, a deficit of fat-soluble vitamins, such as vitamin K, can occur, which determines a deficit of all the factors dependent on this vitamin (factors II, VII, IX, and X; protein C and S), inducing hemorrhagic manifestations or determining excessive vascular coagulation states. There are various gynecological and obstetric complications in this pathology fundamentally linked to all nutrient absorption disorders in the intestine: anemia due to iron deficiency or folic acid deficiency, hypoalbuminemia or hypocalcemia, with the pertinent consequences, such as amenorrhea, infertility, early menopause, spontaneous abortions, and delay in intrauterine growth, taking into account that folic acid, iron, and vitamin K are also essential for fetal organogenesis [61]. The development of arterial and/or venous thrombotic disease and its repercussion at the obstetric level has been linked in CD in most cases to the presence of anticardiolipin and antiphospholipid antibodies as an associated autoimmune pathology [62]. Proteins C and S are vitamin K-dependent factors, physiological coagulation inhibitors that have a short half-life in relation to the other vitamin K-dependent factors, which is associated with their physiological decrease during pregnancy. Thus, an imbalance between procoagulant and physiological anticoagulant factors may occur due to their premature decrease, favoring maternal thrombotic events with important repercussions on fetal development [62].

## 4. Early Gluten Consumption and Development of Celiac Disease

Gluten acts as a trigger for potential CD patients, but although approximately 30% [63] of the world population are genetically predisposed (positive HLA DQ2 or DQ8), the prevalence of the disease is only 1–3% [64]. It is not known what factors act to modify the risk for that 1–3% of the general population. The so-called Swedish epidemic of the 1980s, when there was an increase in CD of up to 4 times greater than expected between 1985 and 1987, set off alarms. The changes in the amount of gluten that infants ingested, as well as the moment in which it occurred, suggested that the time of introduction of gluten or the amount of gluten could act as factors that modified the probability of developing CD. The increase in incidence in Sweden was justified due to the increase in the amount of gluten in malted beverages and cereals and the fact that it was administered after 6 months of age, in addition to being related to the introduction of gluten based on whether or not they were breastfed [22].

In the last 30 years, a multitude of studies have been carried out to determine if there is an optimal time for the introduction of gluten. In 2016, a systematic review with meta-analysis [65] concluded that there is an optimal window for the introduction of gluten, between 4 and 6 months of age, based on the analysis of 15 studies [21,66,67,68,69,70,71,72,73,74,75,76,77,78,79]. Likewise, after analyzing various studies [21,66,69,78], a gradual introduction of the amount of gluten from 4 months was proposed to reduce the risk of developing CD. However, this work does not include two randomized clinical trials [45,80], which are included in the systematic review with meta-analysis by Szajewska et al. [81]. In the study by Lionetti et al. [45], there were no differences in CD diagnoses at age 5 years between the group that received gluten at 6 months and those that received gluten at 12 months. Similarly, in the study by Vriezinga et al. [80], there were no differences in CD diagnoses at 3 years between the group receiving gluten between the group receiving placebo (during 4 and 6 months). Taking into account this, the conclusions of this systematic review with meta-analysis are very different. The authors do not corroborate the previous results, on the contrary, they point out that gluten can be introduced from 4 to 12 months regardless of the risk of developing CD before 5 years, and these results were similar to those of other subsequent systematic reviews [82,83].

After the publication of the previously cited studies [81] and especially after the publication of the two randomized and controlled clinical trials already discussed [45,80], the European Society of Gastroenterology, Hepatology and Nutrition (ESPGHAN) published an update in 2016 of its recommendations [84]. The most important novelties reported were: there is no a recommendation about the capacity of the type of gluten to modify the risk of developing CD; the intake of large amounts of gluten is not recommended during the first months after the introduction of gluten (although there is scarce evidence); and gluten should be introduced between 4 and 12 months of age of the child, omitting the “window of opportunity” previously indicated [85], to prevent the development of CD.

Since then, different studies have been carried out. The TEDDY Study Group published a study in 2016 [86] referring to a case-control study on 2062 births in the study cohort. They observed that gluten intake at the age of 12 months behaved as a risk factor for the development of CD, with CD developing earlier in patients with higher genetic risk, although the amount of gluten was an independent risk factor for the development of CD. The children who consumed more gluten, likewise, had a higher risk than those with the lowest intake (OR: 2.65; 95% CI: 1.70 to 4.13). Subsequently, in 2019, the same group published a new study [87]. They analyzed gluten intake in 6605 children at 6, 9, and 12 months and biannually up to 5 years. Although the increased risk was small, they observed that for every gram of daily gluten added to the diet, there was an increase in CD (HR: 1.50; 95% CI: 1.35 to 1.66).

Recently, Lund-Blix et al. [88] in the Norwegian Mother and Child Cohort Study (MoBa), which included approximately 67,000 children and a mean follow-up time of 11 years, analyzed whether the amount of gluten ingested at age 18 months influenced the development of CD. They observed that the amount of gluten ingested by the children who subsequently presented CD was significantly higher than that of the controls, although an active search for CD was not carried out by analytical determination, but only patients diagnosed after review of the clinical history were included. Although the increased adjusted relative risk (RRa) among those who ate more and less gluten was minimal (1.29, 95% CI, 1.06 to 1.58), the data from this study suggest that the amount of gluten ingested is a factor to consider in the later development of CD.

Likewise, Marild et al. [89] published data from the DAISY study, and 1875 children at risk of CD followed. They observed that for every gram of extra gluten intake, there was an increased risk of CD, although the results were not significant: (HR: 1.04; 95% CI: 0.98 to 1.10). This study, like the study published by Aronsson [87], is limited to a population genetically at risk, unlike the study by Lund-Blix [88], which included children without genetic predisposition.

Unlike previous studies, the study published by Crespo-Escobar et al. [90] from the PreventCD cohort showed that the amount of gluten consumed between 11 and 36 months of age was independent of the development of CD at 6 years old, in contrast with the previous results. However, in this multicenter study, which included several European countries, they did find differences for the HLA-DQ2.2/-DQ7 risk group, observing that a gradual increase in the amount of gluten between 11 and 18 months can act as a modulating factor for this specific group. Later, the data from this cohort in Spain were analyzed [91], concluding that the consumption of gluten during the first 3 years of life does not influence the risk of developing CD before 6 years of age.

## 5. Breastfeeding vs. Infant Formula and Celiac Disease

In recent years, attention has also been paid to the role that exclusive or mixed breastfeeding may play and its role in the prevention of CD. A window of opportunity has been hypothesized in which the mucosal immune response can be modulated, thereby causing different foods to change the probability of developing CD [92].

Human milk is not only a fully adapted source of nutrition for the newborn but also a matrix of immunologically active molecules that modulate the immune response and the susceptibility to developing autoimmune diseases [93]. The World Health Organization (WHO) recommends breast milk as the ideal food for the newborn and infant during the first six months of life, due to its ability to achieve adequate post-statutory growth and maturational development [94].

One of the most important factors in the composition of breast milk is its bioactive agents, which make it have an important immunological effect. It is considered a “functional” food due to the presence of anti-infective agents (lysozyme, lactoferrinand immunoglobulin A), oligosaccharides, anti-inflammatories (polyamines, epithelial growth factor, prostaglandins), and probiotics, among others. Other important components include human milk oligosaccharides (HMOs), which are 10% of the carbohydrates present in human milk and which are practically absent in cow milk. They present a multitude of functions, the main ones being to nourish the infant’s gastrointestinal tract with bacteria [95], since most are not digested and reach the colon, avoiding the adhesion of pathogens, promoting the growth of *bifidobacteria* and *lactobacilli* [96,97], and stimulating the infant’s immune system. They are considered prebiotic agents that modify the infant’s intestinal microbiome [98] (a situation studied in those genetically at risk of CD [99]) and limiting the growth of potentially pathogenic bacteria [100,101]. For all these functions, some studies have linked the ability of HMOs to prevent the acquisition of viral diseases in infants [102].

Human milk also contains a large quantity of microRNAs [103], which are small non-coding RNA molecules that regulate gene expression at the post-transcriptional level, implicated in mechanisms of cell proliferation and also in apoptosis and developmental programming [104,105]. Although its involvement in human milk is unclear, it appears to play an important role in the development of the immune system [106].

Other components, such as lysozyme, also seem to have an antimicrobial effect, thanks to their ability to destroy peptidoglycans of the bacterial wall [107]. Lactoferrin, on the other hand, acts as an iron chelator, and part of the undigested lactoferrin that reaches the intestine inhibits the growth of pathogens, such as *Escherichia coli*, thanks to its ability to compete with said bacteria for ferric iron [108]. Additionally, the milk fat globule membrane (MFGM) is related to the development of the immune system and the functionality of the gastrointestinal tract [109,110]. Viral infections, intestinal dysbiosis, lifestyle, or eating in the early stages of life [111,112,113,114] have been hypothesized to be triggers of CD. The capacity to decrease viral infections is the theoretical basis of the thinking that breastfeeding could decrease the development of CD, due to its antimicrobial capacity.

On the other hand, concomitant elevation of anti-transglutaminase antibodies and elevation of antibodies against cow milk have also been observed in certain patients [115], due to states of increased intestinal permeability, such as in situations of allergy to cow milk proteins [116]. Despite this, some authors have hypothesized that cow’s milk could act as a trigger for CD, due to the presence of advanced glycation end products (AGEs) that are the result of the join of powdered milk and other products, producing a proinflammatory effect with increased intestinal permeability (situation observed in vitro [117]), and as a result of an alteration of the intestinal microbiota [118].

Likewise, the presence of antigliadin antibodies in breast milk has led some authors [119,120] to suggest that breastfeeding may have a protective effect on the development of CD due to its ability to modulate the infant’s immune system. As we can see, there are various mechanisms that have been postulated to explain the relationship between breastfeeding and the development of CD.

From a practical point of view, breastfeeding has been related to the prevention of a multitude of pathologies, acting as a protective factor. Thus, it has been associated with a decrease in autoimmune diseases, such as type 1 diabetes, multiple sclerosis, or rheumatoid arthritis [93,120,121,122,123], and there is currently evidence of its role in the development of atopic eczema and wheezing in the first two years of life and in the incidence of asthma in the first five years of life [124]. Furthermore, the beneficial effects of breastfeeding in the development of obesity have been demonstrated, both exclusively [125] and when compared with artificial formula [126], the effect being greater when it is administered exclusively [127,128] and observing a greater association if it is prolonged [128].

Other authors [129] have studied the relationship between the type of breastfeeding and its influence on inflammatory markers in subjects genetically predisposed to CD. In a sample of 170 children, a higher percentage of CD4+ CD25+ and a lower percentage of CD4+ CD38+ was found in children fed at the breast compared to children fed with artificial formula. The increase in CD4+ CD25+ has been related to the decrease in development of autoimmune diseases through the differentiation of regulatory T cells [130], thus suggesting a beneficial profile related to breastfeeding, due to a more mature and more differentiated immune system towards regulatory T cells.

Several studies [21,71,75,77] have described a protective effect of breastfeeding on the development of CD, although most of the studies published to date have not been able to corroborate this protective effect. Other prospective studies [45,66,67,72,80,131,132,133,134,135] have not been able to demonstrate a beneficial effect of breastfeeding with respect to the development of the disease.

Regarding the duration of breastfeeding, four prospective studies [45,67,72,86] analyzed the influence of the duration of breastfeeding on the risk of developing CD, and five case-control studies [21,75,76,77,133] also investigated whether breastfeeding for a longer time had an advantage over a shorter time of breastfeeding. Of all of them, only two studies [75,77] found statistically significant differences between breastfed infants and infants fed with artificial formulas.

Similarly, only retrospective or case studies have shown a protective factor of receiving breastfeeding at the time of gluten introduction [71,75] not being corroborated by the prospective studies carried out [45,66,67,80,132]. The meta-analysis performed by Akobeng et al. [136] reported that breastfeeding had a protective effect on the development of CD, although it must be taken into account that it is a study that includes studies up to 2004, and only six studies of low methodological quality case controls were included.

A meta-analysis by the PREVENTCD Study Group [81] determined in 2015 that there is no relationship between breastfeeding and the development of CD, nor between its duration and the appearance of disease, these recommendations being identical to those of ESPGHAN in 2016 [84]. These data have been corroborated more recently in a systematic review [137], in which the expert committee determines a very weak level of evidence for the claim that drinking breast milk at some point compared to artificial milk protects against development of CD. Likewise, it concludes with respect to the duration of breastfeeding that the evidence is insufficient to draw conclusions, because in many of the studies, the disease developed before the lactation period had ended, and a clear causal relationship could not be established.

Due to the small number of studies and their methodological weaknesses, the majority being of the case-control type, with a small number of samples and without controlling for confounding factors, these results must be taken with caution.

On the other hand, several studies have analyzed the characteristics of powdered formulas and their relationship with the development of CD. Hyytinen et al. [138] analyzed whether there was a difference in genetically predisposed subjects in taking conventional artificial formula or extensively hydrolyzed formula during the first 6–8 months of life, finding no differences in this regard. Segerstad et al. [139] evaluated the risk of developing CD in relation to artificial feeding in genetically predisposed subjects. They analyzed the amounts of powdered milk that infants ingested and observed that there were no differences in the development of the disease depending on the amount of powdered milk they drank, although the follow-up was only 2 years, thus losing the diagnosis of those subjects who developed the disease years later.

The relationship between feeding in the early stages of life has also been studied according to the predisposition of the subject. Welander et al. [140] studied whether the type of diet influenced the development of CD in children of mothers with CD and mothers without the disease, finding no differences regarding the type of diet between the two groups and suggesting that the type of feeding contributes little to the risk of developing the disease in the offspring of celiac patients, being other factors that explain the increased risk of this group and not the type of breastfeeding or feeding in the first months.

## 6. Importance of the Composition of the Intestinal Microbiota and Celiac Disease

The fetus is exposed to some factors because it does not reside in a sterile intrauterine environment; among them, commensal bacteria from the intestine and/or the maternal bloodstream play an important role. They can cross the placenta and colonize the amniotic fluid. Extrauterine life may be influenced by intestinal crosstalk with colonizing bacteria, inducing a modification of the baby’s immune homeostasis, and being able to provide protection against the expression of diseases (allergy, autoimmune disease, obesity, etc.) in the future. Therefore, it is considered that colonizing intestinal bacteria are essential for the normal development of host defenses [141].

In fact, over the past decade, both scientists and clinicians have recognized the importance of bacteria, particularly bacteria that colonize the gastrointestinal tract, in the metabolic and protective function of the host [142]. The intestinal microbiome is essential to the development of the immune system and begins to assemble in utero. As early as the first year after birth, the microbiota has begun to develop into an adult-like pattern, suggesting that environmental influences in infancy may have important lasting effects [143].

Taking into account that most of the population does not present any problem when consuming gluten, even for those with genetic susceptibility, it is clear that there may be factors other than gluten that could be involved in the development of CD. There is evidence that infants at risk of developing autoimmune disease feature differences in regard to the composition of the microbiota compared to infants without the genetic risk. Sellitto et al. [87] reported that at-risk subjects had a higher abundance of *Firmicutes* and a lower representation of *Bacteriodetes* compared to control infants with a non-selected genetic background. At two years of age, a delay in microbiota maturation was observed, while in not at-risk infants, the maturation was complete at one year.

In addition, the presence of anchored bacillary bacteria, not present in healthy individuals, has been observed in the duodenum of children with CD [144], which may indicate the importance of microorganisms in the pathogenesis of CD. There are studies that focus on evaluating the microbiota present in the intestine because CD is a disease that affects this region [113,145].

Through techniques such as fluorescent in situ hybridization (FISH), in duodenal biopsy samples, a reduction in the population of *Lactobacillus* and *Bifidobacterium* and an increase in *Bacteroides* and *Escherichia coli* in children with CD compared with healthy children were observed [146]. In stool samples and duodenal biopsy from healthy celiac children, an increase in *Bacteroides* sp. was observed through real-time PCR and *Clostridium leptum* in celiac children, while *Bifidobacterium longum* was decreased [147,148]. Likewise, the levels of *Escherichia coli* and *Staphylococcus* were higher both in the biopsies and in the stools of children with active CD compared to those who were treated and controls.

On the contrary, there are other studies that, using the denaturing gradient gel electrophoresis (DGGE) technique, indicate a reduction in the diversity of *Bacteroides* in the biopsies of celiac children regardless of the treatment, so that certain species, such as *B. distasonis*, *B. fragilis*, *B. uniformis*, and *B. ovatus*, appeared more frequently in healthy controls than in active and treated celiac, while *B. vulgatus* appeared increased only in healthy controls [149]. Schippa et al. [150] analyzed the composition of the microbiota of the duodenal mucosa of children with CD and healthy children using temporal temperature gradient electrophoresis (TTGE). A greater diversity was observed in the duodenal mucosa of children with CD, presenting a different electrophoretic profile by TTGE before and after treatment.

Sánchez et al. [151] cultured the microbiota present in duodenal biopsies of children with active CD, treated, and non-celiac controls. They concluded that children with an active form of the disease had a higher proportion in terms of the relative abundance of *proteobacteria* vs. non-celiac controls (16.2 vs. 1.4%), while the opposite occurred with *Firmicutes* (73.2% vs. 93.0%). Specifically, *Klebsiellaoxytoca* (7.1% vs. 0%), *Staphylococcus epidermidis* (18.2% vs. 2.8%), and *Staphylococcus pasteuri* (6.9% vs. 0%) were more abundant in active celiac patients than in controls. On the contrary, in celiac children, species of the family *Streptococcaceae*, (41.6% vs. 81.7%) appeared in a lower proportion compared to non-celiac controls. It should be noted that not all the studies analyzed indicated the existence of clear differences between the microbiota of celiac and healthy children. Ou et al. [152] carried out the characterization of the microbiota of the proximal part of the small intestine by sequencing a region of 16S rDNA and found no differences in bacterial populations between biopsies of children with CD and healthy children.

A previous study that compared microbial communities in infants carrying the DQ2 haplotype with infants who did not carry a compatible haplotype suggested that genetically predisposed infants featured early microbiota alterations. Distinct differences between the microbiota composition at one month of age were observed, with infants who carry DQ2 showing a higher abundance of *Firmicutes* and *Proteobacteria* compared to infants without a genetic predisposition [153].

Moreover, some studies that prospectively screened infants with a first-degree family member with CD from birth found that CD develops quite early in life in this risk group, further supporting the notion that early environmental factors may be very important in the development of CD. These studies found that 16% of infants who have a first-degree relative with CD and who carry HLA DQ2 and/or DQ8 will develop CD by age five, most of whom will be diagnosed by age three. These studies further demonstrated that 38% of infants who are first-degree relatives of CD patients and who carry two copies of DQ2 will develop CD by age five [154].

Taking all the findings mentioned above, “Celiac Disease Genomic, Environmental, Microbiome, and Metabolomic Study” (CDGEMM) is ongoing in the United States (USA), Italy, and Spain in order to investigate the role of gut microbiota in CD. CDGEMM is a prospective longitudinal observational cohort study of infants with a first-degree family member with CD that aims to investigate if the time of gluten introduction, microbiota composition, and genetic asset are involved in the loss of gluten tolerance, and identify and validate specific microbiota and metabolic profiles that are mechanistically linked to gut functions and can anticipate a loss of gluten tolerance in genetically predisposed individuals [154].

In addition, it is important to take into account not only the differences in the composition of the intestinal microbiota between healthy children and CD but also its activity, since it can also be altered. Thus, by means of zymography, it has been revealed that celiac patients present a profile of bacterial proteases capable of hydrolyzing gliadin that was absent in healthy ones. It is shown that in addition to the imbalance in the intestinal microbiota, children with CD present a proteolytic activity of different bacterial origin [155].

Among the environmental factors that are able to modify the microbiota, antibiotics could play an important role. Indeed antibiotics exposure could increase CD risk, as a previous study revealed, showing a higher risk of developing CD in children under an early exposure to antibiotics, with an odds ratio of 1.4 (95% CI, 1.27–1.53) [156].

## 7. Conclusions

The increase in prevalence that is occurring in CD in recent years may not be due solely to the improvement in the diagnosis and the increase in the diagnostic of the disease, as there are other factors that help to contribute to this increase. Given the strong environmental burden that seems to act as a modifying factor for the disease, it is logical that attention has been paid to gluten as a key element in preventing the disease. However, based on the studies published so far, it seems clear that there is strong evidence in favor of the fact that the time of introduction of gluten during lactation (between 4 and 12 months) does not seem to modify the risk of developing CD. To understand the keys to the Swedish epidemic, perhaps it is other triggers that could explain the differences in this population and, also, the different prevalences between countries.

Similarly, there is no strong evidence in favor of the protective factor of breastfeeding and the development of CD, although small studies have shown that there may be a relationship and others have shown immunological changes in genetically at-risk subjects. Despite the different hypotheses proposed, it is not clear why breastfeeding would act as a protective factor of the disease. It seems that due to its anti-infective properties, there could be a delay in diagnosis rather than true protection, and its role in metabolic programming remains tobe discovered, which could act more in the long term.

Despite having recognized, in recent years, different mechanisms as possible disruptors in CD, the evidence is very scarce on the role that diet plays in the early stages of life. More studies are needed to determine which factors may increase the risk of developing the disease in a genetically at-risk population, as well as studies that definitively clarify the role of gluten intake in the first year of life. The dramatic increase in the incidence of the disease makes it necessary to actively search for these factors even from prenatal stages, with fetal programming being a factor that can modulate risk. Diet, genetic risk, microbiota, and environmental interaction are possible triggers of the change in tolerance to an immune response to gluten, and the greater knowledge of them will lead to an improvement in the diagnosis of the disease, or its prevention in early stages. The perinatal environment’s influence on the development of CD is still circumstantial evidence. Large-scale cohort studies and emerging scientific concepts, such as epigenetics, may help us establish the role of these factors. 
